# Transfer and Implementation Process of a Good Practice in Workplace Health Promotion

**DOI:** 10.3390/ijerph18105254

**Published:** 2021-05-14

**Authors:** Francisco Ruiz-Dominguez, Ingrid Stegeman, Javier Dolz-López, Lina Papartyte, Dolores Fernández-Pérez

**Affiliations:** 1 Servicio de Promoción y Acción Local en Salud, Dirección General de Salud Pública y Ordenación Farmacéutica, Consejería de Salud y Familias, Junta de Andalucía, 41020 Sevilla, Spain; mariad.fernandez.perez@juntadeandalucia.es; 2 EuroHealthNet, 1000 Brussels, Belgium; i.stegeman@eurohealthnet.eu (I.S.); l.papartyte@eurohealthnet.eu (L.P.); 3 Distrito Sanitario Granada Metropolitano, Servicio Andaluz de Salud, 18013 Granada, Spain; franciscoj.dolz.sspa@juntadeandalucia.es

**Keywords:** health promotion, disease prevention, good practices, intervention, implementation, workplace, occupational safety and health

## Abstract

The procedure developed by the European Joint Action CHRODIS PLUS (JAC+) to transfer and implement good practices from one setting to another was tested in the context of a workplace health promotion good practice identified in the Region of Lombardy (Italy) and transferred and implemented in two organisations in Andalusia (Spain). This article provides a detailed account on how the JAC+ implementation methodology, which included the use of the SQUIRE (Standards for QUality Improvement Reporting Excellence) guidelines, was applied. It offers a practical overview for the uptake of this methodology and of the good practice itself. The account of how this systematic and rigorous implementation reporting model was applied can be of value to those with an interest in workplace health and in the transfer of good practice and implementation sciences.

## 1. Introduction

This article reports on the transfer and implementation of certain elements of a Workplace Health Promotion (WHP) good practice (originated in Lombardy, Italy) in two organisations in Andalusia (Spain), within the framework of the European Joint Action CHRODIS PLUS (JAC+). The JAC+ was a three-year initiative (2017–2020) under the European Commission’s Third Health Programme. It was designed to encourage EU Member States to collaborate and draw on the best practices available across Europe to prevent and treat chronic disease, which are the main cause of mortality and morbidity across Europe and globally [1]. More than forty beneficiaries representing twenty European countries participated in the Joint Action, which focused in large part on transferring and implementing models of good practice in the field of chronic diseases from one setting to another. Twenty-one new interventions were piloted in the context of the Joint Action. Eight of these pilots focused on the transfer and implementation of five good practices in the field of health promotion, covering various stages of the life-course (3 childhood, 1 workplace, 1 older population) into eight new locations across Europe as part of the JAC+ work strand led by EuroHealthNet.

The main risk factors for chronic disease are “hypertension, tobacco use, high cholesterol, low fruit and vegetable intake, overweight and obesity, sedentary lifestyle and alcohol abuse” [2]. Since these factors are preventable, investments in promotion and disease prevention interventions can clearly be an efficient and cost-effective approach to reduce the incidence of chronic diseases. In its work-strand on health promotion, the Joint Action CHRODIS (2014–2017), which preceded JAC+, identified many good policies and interventions in the field of health promotion and disease prevention from across Europe, but they also confirmed that they are being applied in a piecemeal rather than systematic fashion [3]. JAC+ built on this work to identify how and what has proved effective in one setting can be transferred and scaled in another, as an approach to strengthening this field.

In addition to educational and community settings, workplaces are very suitable settings for health promotion interventions, since it is where many adults spend a large percent of their time. The “Luxembourg Declaration on Workplace Health Promotion in the European Union” [4] defines WHP as “the combined efforts of employers, employees and society to improve the health and well-being of people at work. This can be achieved through a combination of: improving the work organisation and the working environment; promoting active participation; encouraging personal development”. Studies across workplaces and countries have already observed that WHP can generate promising results in terms of positive health outcomes, particularly in reducing the spread of general risk, absenteeism, and healthcare costs, and likely annual return of investments [5,6,7,8,9,10,11].

The Joint Action CHRODIS recognised the WHP Programme developed by the Region of Lombardy (Italy) [12,13] as a good practice on the basis of an established set of quality criteria [14]. The practice targets adults at their workplaces and aims to encourage and enable them to adopt healthier lifestyles and behaviours. It adopts an intersectoral approach and, as a public–private network, it is built on partnerships and collaboration with a range of stakeholders relevant to health in the workplace: associations of enterprises, trade unions, and the regional health system. The programme involves the gradual deployment of health-promoting actions in workplaces over the course of a three-year cycle. Under the programme, workplaces gradually carry out a certain number of activities from a pool of sixty that fall under six areas (healthy eating, promotion of physical activity, tobacco control, work–life balance, and welfare, alcohol prevention and sustainable mobility). In the first year, at least two activities within two areas have to be undertaken, and in each following year, another two activities from two different areas have to be added. By the end of the third year, organisations should have implemented the minimum number of actions in all six health promotion areas covered by the programme. An anonymous assessment questionnaire is used in months 1, 12, and 36 to evaluate the intervention and monitor changes in employees’ behaviours in the relevant areas. When participating organisations have completed the 3-year programme with adequate monitoring and evaluation outcomes, they are accredited as a “health-promoting organisation”. In sum, the Lombardy WHP model applies a clear systematic approach that aligns with the strategic guidelines of the European Commission on Corporate Social Responsibility, as well as the wider strategy of the European Innovation Partnership on Active and Healthy Ageing, amongst others.

The features of the WHP Lombardy model attracted the interest of the Spanish Regional Administration in Andalusia, which is the second largest and the most populated region in Spain. This region places a strong emphasis on health-promotion interventions; the Spanish Ministry of Health certified Andalusia for implementing the highest number of best practices in the country in this area [15,16]. WHP is defined in the IV Andalusian Health Plan (2014–2020) [17] and is enshrined in the Andalusian Law on Public Health [18]. A region-wide WHP programme (“PSLT”, the equivalent in Spanish), which is already in place, evolved from a ‘smoke-free companies’ intervention. In addition to smoking cessation activities, it included physical activity and healthy eating interventions, but the programme needed a more comprehensive assessment approach. Moreover, the Andalusian programme found it a challenge to recruit and engage organisations over a longer term. The more comprehensive nature of the WHP model in Lombardy, which engaged a wide range of stakeholders, was elaborated over time and included systemic follow-up and evaluation and a reward system, appealed to the Andalusian Regional Ministry of Health. Therefore, they decided to replicate and test the selected elements of the Lombardy good practice as a pilot study programme in Andalusia.

Other articles in this journal by the JAC+ partnership have elaborated in more generalised ways on the JAC+ implementation strategy and lessons learned from its application when transferring and implementing good practices in various settings [19,20] including the clinical [21]. This article builds on these and provides a more detailed account of how this strategy was applied in the context of the transfer and implementation of the Lombardy WHP programme to Andalusia. The aim of this article is to illustrate how the JAC+ implementation strategy was applied, the facilitators and barriers to this process that were identified before implementation, and how these were leveraged or overcome during this process, to help ensure the success of the initiative in the new context. It will present on the initial results following a nine-month implementation period. These insights can be of use to other policy makers and practitioners that aim to transfer and implement this or other health promotion programmes into new settings. It can also contribute to the evidence base in the field of transferability and implementation research.

## 2. Materials and Methods

All interventions that were transferred and implemented in the context of JAC+ followed the CHRODIS PLUS guideline on implementation strategy [22,23], which included applying an adapted version of the SQUIRE (Standards for QUality Improvement Reporting Excellence) guidelines [24] to report the entire process. This helped to ensure a systemic approach was taken across all pilots that also made it easier to understand the outcomes of each intervention and the factors that led to difficulties and/or were responsible for the success of the new implementation process.

On the basis of this strategy, the process of transfer and implementation of the WHP intervention in Andalusia consisted of the following four stages, which are described in further detail below. First, the leading partners in Andalusia held several teleconferences and met directly with the Italian representatives, coordinators, implementers, participants, and stakeholders of the Lombardy practice. Real-site visits to the primary context were also undertaken to refine and complement the information with the practicalities described by both staff and users. Then, the lead partners in Andalusia undertook a situation (scope) analysis and feasibility assessments (SWOT analyses). Next, they developed an Action Plan, collaborating closely with both the primary (Italian) implementers and the local (target) working groups. Subsequently, the transfer of the practice was carried out and monitored. Finally, the process and results were evaluated using the indicators included in the Action Plan, and the conclusions were reported and shared.

### 2.1. Knowledge Exchange with the Primary Context

At the start of the transfer and implementation process, representatives from the Andalusian Regional Ministry of Health visited the Regional headquarters of the Lombardy Health Department, which is the owner of the Lombardy practice. The purpose of the visit was to get to know the good practice better, to learn directly from the experts and implementers involved, and to strengthen the cooperation with them. Coordinators, implementers, participants, and other stakeholders from the Italian practice provided thorough explanations of the features of the Lombardy WHP intervention. The delegates from Andalusia described their own context and the departing situation. The Spanish delegates also visited two firms in Lombardy (in Insubria and Brianza) implementing the programme to learn more about how it was being put into practice, hearing directly from both the employers and employees.

At this initial stage, the Lombardy Health Department and the Andalusian Regional Ministry of Health discussed the nature of the transfer and implementation process, what elements of the intervention could be adapted to suit the new context, and what elements had to be implemented to maintain the integrity of the original good practice. During the entire transfer and implementation process, Andalusian and Italian leading teams stayed in contact through emails for follow-up and further coordination. In addition, during the implementation stage, bimonthly meetings took place using videoconferencing systems to discuss relevant actions and finding. Progress reports followed each meeting.

### 2.2. SCOPE and SWOT Analyses

The Andalusian leading partners then met with a number of potential organisations that could implement the programme, finally selecting one medium- and one small-sized organisation. These types of organisations are the most prevalent in Andalusia. The first—EMASAGRA (200 workers, 70% male)—is a joint public–private venture based in the city of Granada that manages all processes related to the water cycle for human consumption. The second—CSIF (35 workers, 50% female)—is an Independent Trade Union for Public Officials, which is one of the three largest trade unions at the state level, with headquarters in Granada.

A Local Implementation Working Group (LIWG) was brought together comprising 16 participants with a wide range of profiles, such as health experts and decision makers from the Andalusian Administration, and implementers, employees, and representatives from the participating organisations. The Andalusian Administration led structured discussions to enable the LIWG to define (a) the problem/challenge; (b) the general purpose of the intervention; (c) the target population; and (d) the essential components of the tasks that needed to be carried out.

The LIWG also engaged in a SWOT (“strengths, weaknesses, opportunities, threats”) analysis to identify the internal strengths and weaknesses, as well as external opportunities for, and threats to, the transfer and implementation of selected components of the WHP intervention from Lombardy to Andalusia. The findings were captured in reports (both in Spanish and English) that were distributed amongst those who had participated in the analysis, and to the Italian partners.

### 2.3. Definition and Development of the Pilot Action Plan

An adapted version of the iterative cyclic nature of “collaborative methodology” [25] was applied to draft, define, and develop the pilot Action Plan to guide the WHP interventions in both organisations. This methodology helped to define and describe all the specific objectives, the concrete activities, and the key performance indicators to measure the progress and impact of the interventions. JAC+ implementers were tutored (by staff from the Kronikgune Institute for Health Services and the European Commission) on how to use the proposed methodology. This was done with a series of webinars, guideline documents, and teleconferences. A designated expert of the European Commission revised and approved the Andalusian WHP Pilot Action Plan and its Final Implementation Report. In parallel and subsequently, a plan–do–study–act (PDSA) cycle [19] was used to test interventions and enable rapid assessment and flexibility to adapt the intervention according to feedback.

### 2.4. Evaluation

The success of the transfer and implementation of this WPH programme was assessed on the basis of process indicators and an outcome evaluation. The processes indicators focused on, amongst other things, ensuring the organisations had taken steps to comply with existing workplace health and safety regulations, established a steering group that met regularly, and that staff was participating on a regular basis in the WHP activities. Outcomes were evaluated on the basis of pre–post assessment questionnaires (translated from the Italian) that focussed on health data, health-related habits, and the usefulness of the intervention.

In order to link answers with respondents before and after the intervention, and to enable some of the subsequent analyses, participants were asked to use as an identifying variable the last 5 digits and the letter of their national identification number. Changes in weight and waist circumference were measured as continuous variables. For example, the questionnaires included questions on the frequency and intensity of physical activity, sweet or tobacco consumption, or daily intake of fruits and vegetables. Finally, they asked participants to rate the usefulness (or applicability) of the activities they took part in (along a 4-point scale ranging from “not useful” to “very useful”). Satisfaction surveys were distributed amongst all participants in the WHP workshops. Additionally, follow-up meeting and frequent communication with members of the steering group in each organisation were maintained during the implementation process, and semi-structured final evaluation interviews were conducted with most of the members involved in the LIWG from each organisation.

## 3. Results

The results of this study will be presented in three parts. The first part sets out the outcomes of the SWOT analysis and how this was incorporated to form the basis of the Action Plan to implement the WHP Lombardy model in Andalusia. The second sets out the evaluation outcomes of the initial nine months of the intervention. The third part sets out the main barriers and enablers to the process, and the lessons learned.

### 3.1. Uptake of the WHP Lombardy Model in Andalusia

One of the key differences between the two contexts identified from the outset of the project was that the Lombardy WHP programme operated in a setting with big-sized organisations with a large number of workers. These organisations had large human resource departments, and/or medical and occupational health services that were able to provide assistance on health issues. However, in Spain´s Southern region of Andalusia, the majority of companies are small or medium sized, and they find it more difficult to carry out certain types of interventions themselves. Therefore, support from the Public Administration is vital when it comes to WHP here, and a team of around 50 health promotion experts throughout Andalusia is providing support to the WHP programme, training the workforce (on a voluntary basis free-of-cost for the organisations) or helping the organisations to carry out the activities and workshops. Therefore, it was clear that the Public Administration in Andalusia would play a much more active role in implementing the WHP programme than in Lombardy, where Local Health Agencies played a supervisory role but did not provide trainings or run activities directly.

The initial situational analyses obtained from the structured group discussions, carried out at the outset (see Section 2.2), identified the internal strengths and weaknesses as well as external opportunities and threats to the transfer and implementation of the WHP good practice in Andalusia (SWOT). Amongst the strengths identified were the Public Administration’s previous experience in the field (PSLT programme), which could be applied to provide training and support to participant organisations; the commitment of the implementing organisations’ managerial staff, and the fact that they had fluid communication systems in place between managers and their employees. Amongst the opportunities identified were the possibility to evaluate the initiatives; the accreditation process, which could motivate companies by boosting their social corporate image and the social or institutional endorsement of WHP activities. Amongst the weaknesses and threats identified were the possible reluctance of the workforce to participate in company-run WHP activities and the lack of personnel with the necessary skills to support the activities. The lack of a general culture, or awareness of, or interest in health promotion in general was also perceived as a threat to the implementation. The matrix with the most important categories of selected factors from the SWOT analysis is presented in Table 1.

The LIWG established what they considered to be the priority areas for strategic action. These are shown in Table 2. It was apparent from the outset that they considered the following factors as key to the success of the implementation process: the involvement of high managerial leaders; the participation of the workforce; support from the Public Administration; obtaining good examples of the kinds of actions that could be undertaken; and applying good communication channels.

The implementation leads in Andalusia considered the core actions that needed to be taken to ensure the integrity of the Lombardy WHP model and compared these to the SWOT analysis. This led them to the following five categories of actions that formed the basis of the Pilot Action Plan.

1. Creation of a steering group. A steering group comprised of managers, employee representatives, risk prevention, and/or human resources professionals was established to ensure an efficient design, implementation, and evaluation of the intervention in each of the organisations. It was responsible for the implementation of the Action Plan within the respective organisations.
2. Ensuring regulatory compliance. This was an essential preliminary step in the Lombardy WHP model. To ensure a sound basis for the implementation of any further WHP actions, organisations had to show that the required measures on social security; workplace and environmental safety; occupational health and risk prevention were all correctly in place.
3. Pre and post implementation questionnaires. They were conducted during the initial and final months of the actual implementation of the WHP actions (i.e., at the beginning and after the initial period of nine months). The Andalusian School of Public Health was responsible of the technicalities of the online survey as well as the analyses of the data.
4. Promoting employee participation. Organisations had to encourage the participation of the workforce in the planned activities. To do this, they involved key representatives (such as managers, risk prevention, and human resources professionals) in the following tasks: presenting the WHP programme to all employees; disseminating information about the selected activities via relevant communication channels; implementing the activities; encouraging and facilitating employees´ participation in the activities, preferably during working hours.
5. Developing a WHP certification system in line with Lombardy´s continuity and award system.

Then, the steering groups coordinated a process whereby each organisation could select what specific health promotion activities would be provided in the first year. EMASAGRA selected the area of “Physical activity”. The activities selected in relation to the first area involved improving opportunities to do physical exercise, mainly by means of setting up a health and exercise hall accessible to all workers; encouraging the use of stairs; and sponsoring the “PUMP” intervention in Andalusia that involves the establishment of walking groups that aim to accomplish the goal of taking one million steps together [26]. They also selected the area of “work–life balance” and introduced three activities in this area: adopting a flexitime policy (allowing margin for employees to alter workday start and finish times); smart working (offering the possibility of tele-working to all employees); and providing a “city-pack” system (hub locker inside the premises of the organisation to collect personal packages with a unique pick-up code).

In turn, CSIF focused on “healthy eating” by making fruit and/or fresh seasonal vegetables available for employees 2 days a week and conducting small-group workshops providing practical advice on healthy nutrition. They initially thought of conducting smoking-cessation groups, but the plan was postponed for a year because the number of participants needed to start these groups was not reached. In exchange, they adopted the “PUMP” intervention and sustainable mobility. All activities were run on a voluntary basis and free of cost to employees.

The steering groups in each organisation further defined and broke down these five overarching categories in their Action Plans into more specific objectives, associated actions, timeframes, and process and outcome indicators. This led to the development of a final Action Plan with specific objectives and key performance indicators across the five areas. The Action Plan is included in Table 3.

Both organisations reported implementing the activities to the Regional Ministry of Health in written form and audio-visually and confirmed what actions they were planning to implement in the following period.

### 3.2. Evaluation Outcomes

Following the first nine months of implementation, many of the indicators of success had been met. All five selected features of the Lombardy WHP model were successfully implemented by the two participant organisations in Andalusia. In addition, regulatory compliance was certified; steering groups, endorsed by top managers, were established; and health-promotion activities were efficiently carried out, with the guidance and support of the Public Administration and the proactive contribution of the organisations. The majority of workers participated in the WHP activities (for example, 85% of the CSIF employees and 62% of the EMASAGRA employees attended the introductory sessions held). Personnel unable to attend the sessions (because e.g., these did not correspond to their working hours, or due to other commitments) received the necessary information from a colleague trained for this purpose. Both organisations ran in-house information campaigns to raise awareness about existing and upcoming activities and to encourage participation. The workforce participated in the activities on a voluntary basis and recognised their value. 

Despite the relatively short initial period, positive changes related to healthy eating or physical activity could be observed in the first data analyses following the first nine months of intervention. Pre- and post-implementation tests of the first intervention were conducted anonymously. Organisations provided all the necessary conditions for the workforce to respond the questionnaires online. In very few cases when this was not possible, printed copies were provided. More than 50% of the personnel of each organisation completed the questionnaires (see Table 4). Amongst them, 74 participants (who completed both pre and post questionnaires) across both organisations were paired. Two types of analyses were carried out: paired sample *t*-tests for continuous variables (see Table 5), and percentage comparisons for descriptive tendencies and qualitative variables.

In general, statistically significant changes were not yet observed when analysing the data from before and after the first nine months of intervention. However, when carrying out organisation-segmented analyses comparing pre–post percentage differences, positive tendencies became apparent in certain variables. In the group of respondents from EMASAGRA, there was a decline in the frequency of sweets consumption (4 or 5 times/week: 10.8% vs. 4.6%), and in the absence of physical activity (from 70.8% to 58.5%). There was also an increase in the percentage of people declaring a daily practice of some physical activity (from 23.1% to 35.4%). Some positive changes related to healthy eating and physical activity were also observable at CSIF, although the total number of participants that completed both questionnaires was very small, making comparability difficult.

Apart from that, questionnaire items related to the appraisal of the intervention reflect an increase in the percentage of people who considered participating in the WHP actions “very useful”, most notably in relation to the areas of “physical activity” (CSIF: 11.1% to 77.8%; EMASAGRA: 44.6% to 58.5%) and “healthy eating” (CSIF: 11.1% to 88.9%).The results of evaluation surveys of the WHP workshops reflected a very high level of satisfaction from being involved in the intervention (with, for example, 100% of participants at the CSIF workshops on healthy eating indicating they were satisfied with the information received; 76.2% of them indicated they were ‘very satisfied’ and 23.8% ‘quite satisfied’). Interviews with members of the LIWG in each organisation confirmed this too.

### 3.3. Barriers, Enablers, and Lessons Learned

The Andalusian team presented full details of their activities and their outcomes, complying with the JAC+ guidelines, in a comprehensive Implementation Report [27]. As part of this structured reporting, the members of the implementation group reflected upon the barriers and enablers of the implementation and offered suggestions for future endeavours across five dimensions: (1) sustainability, (2) organisation, (3) empowerment, (4) communication, and (5) monitoring and evaluation. 

Some of the potential weaknesses and threats identified at the outset of the implementation process, such as a lack of a culture around, or appreciation for health promotion did not emerge as a problem. Other potential weaknesses and threats did materialise; for example, there was a lack of trained staff with the time available to deliver the programme. In addition, employees’ schedules made it difficult for them to participate in different activities. Some were also reluctant to participate in company-run activities and to provide private information concerning their life habits in questionnaires that were initially perceived as long and cumbersome. 

The support and guidance provided by the Regional Ministry of Health, which was free of charge, contributed to overcoming these barriers. So did the fact that the programme was endorsed at the managerial level, who allowed staff to dedicate working time to participate in the programme. Implementers also found that an important element of success lay in the resources dedicated to building the capacities of people in each respective organisation. As a result, they became qualified disseminators who amplified the effect of the training and guidance provided by the Andalusian Administration and helped raise awareness and knowledge of other people (train the trainers approach). The employees that were trained took the lead in rolling out the programme further. A full overview of the barriers identified, how they were overcome, and suggestions for future implementers is available in Table 6.

All stakeholders involved considered the practice of continuously eliciting spaces for information and knowledge exchange, formal and informal follow-up, consultation, and feedback as vital to the transfer and implementation process. For example, during the implementation stage, the lead JAC+ partners in Andalusia informed their Italian counterparts of how the process was evolving. In turn, the Andalusian leaders were in constant contact with the LIWG representatives from each organisation. Consistent communication and dissemination through a range of channels (newsletters, posters, announcements, as was the face-to-face sessions, workshops, and informal contacts) was vital to engage key target groups.

The role of the designated steering group to guide and refine actions and celebrate achievements also facilitated dissemination and adherence to the actions. Finally, because the LIWG had already considered, anticipated, and discussed potential challenges in early stages of the intervention, they were better prepared to address them if they arose.

An account of the transfer and implementation of this good practice and the lessons learned is also incorporated in the overview report presenting and analysing the results of all five good practices transferred and implemented in the context of JAC+ [28]. The Italian partners were also pleased with the way the practice was transferred, as reflected in various written minutes of the teleconferences held.

## 4. Discussion

To our knowledge, this is the first comprehensive article that presents the entire process of the JAC+ implementation strategy fully illustrated with details and results from the transfer and implementation of a selected good practice from a primary to a new context. This article has set out how the WHP model from Lombardy was piloted in two workplace settings in Andalusia. The broader contexts in these two locations differed in that companies in Lombardy tend to be larger, with ‘in house’ human resource departments that can manage the programme, while companies in Andalusia tend to be small, with little or no existing capacities in this area. Therefore, support from the regional Ministry of Health in Andalusia and from managerial structures in both participating organisations was considered essential to the success of the pilot implementations. Following the first nine months of the pilot implementations in Andalusia, the results of JAC+ reflected that interventions had ‘taken root’ in their new contexts. The onset of the COVID-19 crisis, shortly after the initial nine months, has complicated the further implementation of the two pilot activities, but most of the activities have continued as planned. The intervention of corporate group walks “PUMP—For a Million Steps” was the only one that had to be halted due to COVID-19 measures. Interestingly, the smart-working measures that were introduced for all employees by EMASAGRA during the implementation process (before the COVID-19 crisis), proved very beneficial after the outbreak of this crisis, as these measures were extended to most employees for several months in a row. The “tobacco cessation” activities that could not be started in the first year began in the second year. The minimum number of people that are necessary to constitute a group (10 people) was reached when the company allowed some relatives of employees to take part. Family members of employees from both organisations (CSIF and EMASAGRA) also participated in the “PUMP” challenge, to the greater satisfaction of all involved. This confirms that involving family members in workplace health programmes can be beneficial to their expansion. That was a practice already present in the Andalusian WHP programme (PSLT). By including family and (close) acquaintances, WHP can help to ‘bridge’ personal, or home and work life, promoting more integrated and ‘holistic’ approaches to health and well-being.

The other area from the Lombardy model that is pending implementation is “alcohol prevention”, as it exceeds the competence of the Health Administration in Andalusia, but arrangements are being made to pilot activities in this area too. As such, the activities taking place in the two organisations now cover five of the six health promotion areas of the Lombardy model.

The lead implementers attribute the initial success of the pilot interventions to application of the JAC+ implementation strategy. The features of the strategy that they found particularly important were that it called for them to (1) bring together, from the outset, a team comprised of a variety of profiles, sectors, and experience levels—the LIWG; (2) carry out situation and SWOT analyses; (3) develop an Action Plan and operationalising the actions through measurable indicators; and (4) collect data to test and analyse the intervention. According to the Andalusian implementers, co-creating a clear Action Plan ensured a shared vision and common goal. It served as a source of inspiration and motivation to all the members of the implementation teams. The importance of using a clear implementation framework was in fact highlighted by all teams that transferred a good practice intervention in the area of health promotion and disease prevention in the context of JAC+ [28]. Another JAC+ study that explored the success factors and barriers to intra and intersectoral collaboration in health promotion and prevention also drew this conclusion [29]. The Andalusian implementation team also considered the adherence to the SQUIRE (Standards for QUality Improvement Reporting Excellence) guidelines as important to the success of the pilot interventions, since this required all partners to document each step in a detailed manner, which supported technical robustness, team reflection, and the quality of the outcomes. While COVID-19 will affect some of the actions planned for the subsequent years of the pilot, the experience that the LIWG and steering members gained in anticipating and resolving potential challenges during the intervention (such as overcoming the inability to participate in face-to-face activities), prepared them to address the unexpected challenges.

Despite the difficulties posed by COVID-19, both organisations maintain their commitment to continue to implement the model. The fact that the costs of the programme are fairly low to the companies involved in Andalusia, requiring above all the commitment of managerial support and investments in staff time, facilitated the continuation of the programme in both companies. The Regional Ministry of Health has the resources to provide free training. Carrying out, facilitating, and/or supporting the health-promoting activities in the companies was also essential to its success. This was a contextual strength in Andalusia that could be leveraged to help ensure the success of the model there. Once the three-year implementation period is successfully finished, the Regional Ministry of Health in Andalusia will accredit the two companies as “health-promoting” organisations in a public event. The event, which will be covered by regional media, will publicise the WPH programme, triggering the engagement of more firms and strengthening the profile of the participating organisations as ‘socially responsible’.

Based on their experience in the field of WHP, which has been strengthened through the process of transferring and implementing this intervention, the Andalusian Regional Ministry of Health is convinced of the value of investing in this field and in the process of gradually but consistently building capacities within the organisations to deliver the WHP programme. New firms with different profiles (new sectors and sizes) are already expressing interest, on the basis of information about the pilot and the model that has been incorporated on the Regional Ministry of Health website. If results after completion of the pilot is confirmed to be positive, it will upgrade its current WHP Programme, PSLT, presumably renaming it PSLT+ in line with the Lombardy WHP model and the JAC+ implementation strategy. This will mean all organisations in Andalusia will be able to introduce new activities over time, and have access to the systemic follow-up, evaluation, and reward system. The Regional Ministry will aim to engage more organisations by publicising the programme via press notes, public appearances at events, and merchandise used by participant organisations. This will enhance the visibility and motivation of those organisations already involved. They, and all new organisations that agree to take part, will have the possibility to join a network, or collaborative structure, of all organisations involved in the programme, to enable them to share experiences.

While this experience of transfer and implementation can, to date, be regarded as successful, it also reflects that this process is by no means an easy one, and that success cannot be taken for granted. Identifying potential weaknesses, threats, and barriers to implementation in each new context from the outset is essential to anticipate problems and how they can be addressed. Taking the time for this, to understand the complexity of the systems into which new initiatives will be introduced, can be time-consuming. Defining the objectives, actions, designing a Pilot Action Plan, collecting data, and reporting all stages and details in a structured standardised way also require time. Time constraints and the saturation of qualified staff (or their absence) can affect the motivation of implementers to willingly assume some tasks. A commitment to this process, and close institutional guidance and support, as provided in this pilot study, is essential. However, the initial investments in time and resources eventually pay off, as this provides new initiatives with sound foundations from which they can then be scaled. Therefore it is crucial to continue identifying and highlighting the benefits of the investments to the people and organisations involved, and to society at large, in terms of improving health and well-being and reducing healthcare costs.

As people across the world have been battling the pandemic resulting from a new communicable disease, awareness has risen of the importance of health promotion and disease prevention in strategies to contain the spread of the virus and its impact. Investing in health promotion and disease prevention can yield significant returns, as there is a substantial evidence base suggesting they are cost-effective [30]. This is a reason why interventions such as this one remain so important, and why the European Commission is encouraging greater investments in the field and the take up of more good practices such as this one. The Joint Action CHRODIS (2014–2017), which preceded JAC+, developed a range of criteria to assess and identify good practices in the field of health promotion and disease prevention [14]. The European Commission has subsequently adopted these criteria to evaluate public health practices and collect them in a ‘Best Practice Portal’ [31]. The portal allows for a common mechanism to identify, validate, and exchange good practice in public health and makes evaluated good practices widely available. Awakened to the need to strengthen health systems across Europe, and collaboration between them, the European Union officially launched the ambitious ‘*EU4Health Programme*’ in March 2021, with a budget 12 times bigger than its previous health programme [32]. The regulation mandates that 20% of the budget be spent on action in the field of health promotion and disease prevention to address risk factors such as obesity, unhealthy diets, and physical inactivity. One of the indicators for the evaluation of the programme will be the number of Member States implementing best practices in the field of health promotion.

The detailed account of transfer and implementation of a good practice reported in this article is very timely to contribute to a better understanding of how the process of transferring and implementing a best practice can be effective. The SQUIRE approach and JAC+ implementation methodology can serve as very useful tools in this process. The CHRODIS PLUS Implementation Guidelines [22,23] provide a suitable standard to guide the successful transfer and implementations of health promotion and disease prevention interventions. Implementers of good practices within and across European countries, such as those included in the European Public Health Best Practice Portal, can share their documented experiences using the JAC+ framework, to contribute to a more systematic replication of good practices and strengthen this field of inquiry. The use of this framework ensured that all the knowledge was well captured, so that others interested in transferring and implementing the intervention could benefit from this learning.

## 5. Conclusions

The implementation strategy and approach developed by the JAC+ was trialed and tested in the context of a WHP good practice identified in the Region of Lombardy (Italy) and transferred and implemented in Andalusia (Spain). The main objectives of the implementation were successfully achieved: (1) compliance with relevant regulations was certified, WHP activities were satisfactorily carried out and planning for future engagement was also confirmed; (2) all parties were involved from the beginning and the workforce actively participated in the WHP actions; (3) healthy behaviours and awareness were enhanced in employees and they clearly valued them as very useful.

This example reflects how an initiative in the field of health promotion and disease prevention that has proved effective in one setting can be transferred, to strengthen health systems and outcomes in another setting. If the pilot study in Andalusia proves effective once it has completed its full three-year cycle, the Regional Ministry of Health will ‘upgrade’ its current WHP on the basis of the model. However, the success of such a process cannot be taken for granted, since a wide range of contextual factors can interfere with this. Strong outcomes are more likely if implementers apply, from the outset, a rigorous implementation strategy or framework, such as the one designed by JAC+. This strategy required the new implementers to bring together an intersectoral team (LIWG) to consider the factors, both positive and negative, that can influence the implementation process, to develop a comprehensive action plan, and identify indicators of success. This article has demonstrated what this process looks like in practice.

Since the initial nine months of this pilot study was completed, the COVID-19 pandemic has increased awareness of the need to invest more in health promotion and disease prevention. The European Commission is encouraging the identification and exchange of best practice as a key approach to strengthening this field. The value of this approach will depend upon the extent to which the field of ‘implementation science’ is taken seriously and developed further. This article has made a contribution to this field. More research and investments are also needed to demonstrate the cost–benefits of such actions to demonstrate value and to motivate more professionals to get involved.

## Figures and Tables

**Table 1:** Results of SWOT matrix by the Local Implementation Working Group identifying key priority areas for strategic action.

**Strengths**	**Weaknesses**
Previous experience in WHP Managerial involvement Ongoing training Communication systems for employees	Low participation of professionals in the company-run activities
Availability of support and resources	Lack of trained personnel
**Opportunities**	**Threats**
Evidence of health outcomes Institutional recognition	Consideration of Health Promotion as a low-level intervention
Commitment and support to WHP interventions	Healthcare approach vs. Health Promotion.

**Table 2:** Areas prioritised by the Local Implementation Working Group, where strategic action was considered most needed, ranked in order or relevance.

Prioritised Areas	Ranking
Managerial involvement in WHP interventions	1
Enhancing employee’s motivation to participate in HP sessions and activities	2
Guidance and support from the Public Administration	3
Collecting specific examples (“community of practices”)	4
Enhanced communication via new or existing channels	5

**Table 3:** Main outcomes of the Pilot Action Plan (after 9 months).

**General Objective:** To implement elements of Lombardy’s JA CHRODIS Good Practice “Workplace Health Promotion” in the Andalusian Strategy of Health Promotion at Workplaces.	**Key Performance Indicators (KPI)**			
	**Process**	**Outcomes**		**Sources of Information**
		**Baseline (Pre Questionnaire)**	**Current Value (Post Questionnaire)**	
**Specific Objective 1 (SO1)**: To ensure organisational endorsement of WHP.				
**Activities SO1**: Certifying that the organisations are aware and take the steps to comply with regulations relevant to: Health Promotion, Social Security, Workplace and Environmental Safety. Efficient starting and functioning of a Steering Group in each participant organisation.	**1.2**: Certified compliance in all areas specified. **1.2**: ≥ 2 steering group meetings/communications per month **1.3**: >2 attendees to the steering group meetings	N/A	N/A	CSJA: ✓ Original certifying documents. ✓ Listing of message communications and meetings (calls and minutes).
**Specific Objective 2 (SO2)**: To encourage workforce participation in the WHP actions.				
**Activities SO2:** Ensuring the majority of workers participate in the WHP activities. Conducting small group sessions to deliver the messages in a practical way. *Pre & Post Assessment Questionnaires* (health data, health-related habits, usefulness of intervention).	**2.1**: % employees attending the sessions: *E: 62%; C:85%*. **2.2**: Number of introductory sessions: *E: 12; C: 2.* **2.3**: >50% employees responded pre-post assesment questionnaire	**2.4**: (sample highlighted items) ➢ E: 23% physical activity almost everyday. ➢ E: 11% participants comsume sweets 4-5 days/week. ➢ C: 11% participants regard healthy eating activities very useful. ➢ C: 11% participants regard physical activities very useful.	**2.4**: (positive differences) ✓ E: 35% participants do physical activity almost everyday. ✓ E: 5% participants comsume sweets 4-5 days/week. ✓ C: 90% participants regard healthy eating activities very useful. ✓ C: 78% participants regard physical activities very useful.	✓ Pre&Post: EASP Analyses. ✓ Sessions attendance lists. ✓ SS: CSJA Corporate Information System.
**Specific Objective 3 (SO3)**: To enable the continuity of the engagement of participating organisations.				
**Activities SO3**: Accredit correct implementation and planning of continuation. Institutional certification of the correct implementation of actions (in line with Lombardy´s WHP Model).	**3.1**: ≥ 2 good practices in two different intervention areas.	N/A	**3.2**: Existing 2-year planning of continuation with ≥ 2 new good practices per year/per organisation.	CSJA: ✓ Reports of activities and meeting minutes. ✓ WHP certification.

Legend: C: CSIF; CSJA: Andalusian Regional Ministry of Health and Families; EASP: Andalusian School of Public Health; E: EMASAGRA; SS: Satisfaction survey.

**Table 4:** Response rates to questionnaires and paired respondents.

Participant Organisation	PRE-Implementation Questionnaires	POST-Implementation Questionnaires	PRE-POST Paired Respondents
**EMASAGRA**	113 (57%)	119 (60%)	65
**CSIF Granada**	33 (94%)	20 (57%)	9
**TOTAL**	146	139	74

**Table 5:** Weight and waist circumference paired sample analyses (Student’s *t*).

Organisation	Continuous Variables	Mean	*n*	Standard Error	*t* -Statistic	Degrees of Freedom	*p*-Value
EMASAGRA	Weight 1	75.423	65	1.7703	−0.612	64	0.543
	Weight 2	75.658	65	1.7752			
	Waist circumference 1	82.404	28	5.0807	1.142	25	0.264
	Waist circumference 2	82.8525	28	4.10940			
CSIF Granada	Weight 1	74.222	9	3.0174	0.577	8	0.580
	Weight 2	73.556	9	3.0327			
	Waist circumference 1	84.000	8	4.3956	−0.786	6	0.462
	Waist circumference 2	76.6250	8	11.59270			

**Table 6:** Barriers, enablers, and suggestions for future implementations.

	Barriers	Enablers	Suggestions
**S**	Lack of workplace health promotion culture and knowledge (exclusive healthcare-centred approach).	Strong institutional support, close guidance, and capacity building (free-of-cost, in each specific workplace).	Share a WHP long-term vision. Receive support from and be accompanied by the Public Administration. Allocate flexible but sustained resources.
**O**	Scarce structural resources. Workforce reluctance to participate in company-run activities and to provide information concerning their life habits. Implementation difficulties, related to the characteristics of each organisation (e.g., night-shifts, attention to the public, etc.) and employees´ daily tasks and agendas.	Managerial endorsement and workforce involvement from the beginning. Training of trainers provided by experts. Availability of structural resources (work hours, dedicated personnel, some funding—optional) Adaptation to different times and shifts.	Involve all parties from the beginning: managerial level, organisational leaders, workforce representatives, human resources, occupational and risk prevention professionals, etc. Plan and define a WHP systematic uptake embedding WHP within the organisational long-term health-related plans and strategies.
**E**	Lack of trained personnel, particularly in the case of small and medium-size organisations.	Adherence to a clearly defined systematic approach. Broader WHP awareness. Availability of standard documents and guidelines.	Development of legislation with clear-cut indications. Subsidies and aids (tax allowances, agreements, etc.) to enforce WHP implementations.
**C**	Geographic dispersion of centres. Difficulty or impossibility to participate in face-to-face activities.	Exposition to different communication channels (newsletters, posters, announcements, etc.). Face-to-face general sessions, workshops and informal channels of communication.	Gradual but constant capacity building of key personnel and disseminators. Enhance visibility via new or existing channels and formats. Building upon pre-existing collaborative structures prompts mutual support and networking.
**M**	Long cumbersome questionnaires.	Steering group meetings to refine any necessary action or to celebrate short-term achievements.	Document all steps through. Collect evidence and indicators (obtain support from experts).

S = Sustainability; O = Organisation; E = Empowerment; C = Communication; M = Monitoring and Evaluation.

## Data Availability

The data presented in this study are available on request from the corresponding author. Some data was obtained from third parties and are available from the corresponding author with their permission.
